# Inference for epidemic models with time‐varying infection rates: Tracking the dynamics of oak processionary moth in the UK

**DOI:** 10.1002/ece3.8871

**Published:** 2022-05-02

**Authors:** Laura E. Wadkin, Julia Branson, Andrew Hoppit, Nicholas G. Parker, Andrew Golightly, Andrew W. Baggaley

**Affiliations:** ^1^ 5994 School of Mathematics, Statistics and Physics Newcastle University Newcastle upon Tyne UK; ^2^ 7423 GeoData, Geography and Environmental Science University of Southampton Southampton UK; ^3^ Forestry Commission England Nobel House London UK; ^4^ 3057 Department of Mathematical Sciences Durham University Durham UK

**Keywords:** Bayesian inference, epidemics, Markov chain Monte Carlo, oak processionary moth, SIR model, stochastic differential equation, susceptible‐infected‐removed model

## Abstract

1. Invasive pests pose a great threat to forest, woodland, and urban tree ecosystems. The oak processionary moth (OPM) is a destructive pest of oak trees, first reported in the UK in 2006. Despite great efforts to contain the outbreak within the original infested area of South‐East England, OPM continues to spread.
2. Here, we analyze data consisting of the numbers of OPM nests removed each year from two parks in London between 2013 and 2020. Using a state‐of‐the‐art Bayesian inference scheme, we estimate the parameters for a stochastic compartmental SIR (susceptible, infested, and removed) model with a time‐varying infestation rate to describe the spread of OPM.
3. We find that the infestation rate and subsequent basic reproduction number have remained constant since 2013 (with $R_{0}$ between one and two). This shows further controls must be taken to reduce $R_{0}$ below one and stop the advance of OPM into other areas of England.
4. *Synthesis*. Our findings demonstrate the applicability of the SIR model to describing OPM spread and show that further controls are needed to reduce the infestation rate. The proposed statistical methodology is a powerful tool to explore the nature of a time‐varying infestation rate, applicable to other partially observed time series epidemic data.

## Graphical abstract

The oak processionary moth is a destructive invasive pest. Here, we use state‐of‐the‐art Bayesian inference techniques to estimate the infestation rate of the moth in London parks between 2013 and 2020.

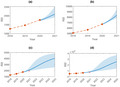

## 1 INTRODUCTION

Invasive pests, such as non‐native insects, pose a threat to forest, woodland, and urban tree ecosystems by damaging and killing trees and reducing biodiversity (Freer‐Smith & Webber, 2017; Kenis et al., 2009; Manchester & Bullock, 2000). This threat has increased in recent years due to growth in international travel and trade (Roy et al., 2014) coupled with a changing climate driving the migration of species into new ecosystems (Lenzner et al., 2020). The loss of biodiversity has a profound economic impact, through short‐ to long‐term control measures and the impact on ecosystem services (Aukema et al., 2011; Boyd et al., 2013; Bradshaw et al., 2016).

The oak processionary moth (OPM), *Thaumetopoea processionea*, is an invasive and destructive pest of oak trees, causing defoliation and making trees vulnerable to other stressors and pathogens. The larvae of OPM have poisonous hairs, containing an urticating toxin (thaumetopoein) which is harmful to human and animal health (Gottschling & Meyer, 2006; Maier et al., 2003, 2004; Rahlenbeck & Utikal, 2015).

OPM was introduced to the UK through accidental imports on live oak plants, first reported in 2006. Up to 2010, the governmental policy was one of eradication (Mindlin et al., 2012; Tomlinson et al., 2015). However, in 2011 it was decided that OPM was fully established in the South‐East England area and so the government moved to a containment strategy, aiming to contain the OPM infestations within this original outbreak area (Tomlinson et al., 2015). In 2018, legislation was introduced to curb continuing imports through the Plant Health Order (Plant Health (England) (Amendment) (no. 3) Order, 2018). Despite the containment strategies, the extent of OPM has continued to spread with recent analysis suggesting an expansion rate of 1.7 km/year for 2006–2014, with an increase to 6 km/year from 2015 onwards (Suprunenko et al., 2021). The regions surrounding the current infestation area are particularly climatically suitable (Godefroid et al., 2020), and so being able to predict and control the future dynamics of the OPM population is crucial to protect these areas.

Mathematical models provide a powerful tool for describing and predicting the spread of tree disease and pest infestation (Gertsev & Gertseva, 2004; Orozco‐Fuentes et al., 2019; Wang & Song, 2008). For OPM, previous work has included using models from electric network theory to predict high‐risk regions (Cowley et al., 2015) along with species distribution models to examine the spatial distributions of OPM (Scholtens, 2021) and the effects of climate change on its expansion (Godefroid et al., 2020). Bayesian inference can be used to inform and evaluate these ecological mathematical models (Ellison, 2004). Previously, Bayesian approaches have been used to estimate key parameters in the spatio‐temporal invasion of alien species (Cook et al., 2007); however, the techniques have yet to be applied to data for the spread of OPM. Nevertheless, the Bayesian paradigm provides a natural mechanism for quantifying and propagating uncertainty in the model parameters and dynamic components. Consequently, Bayesian inference techniques have been ubiquitously applied in the broad area of epidemiology (see e.g., Fuchs, 2013; Kypraios et al., 2017; McKinley et al., 2014 for an overview).

In this paper, we use data tracking the numbers and locations of OPM nests removed from oak trees as part of a control program in two parks in London. We illustrate the use of statistical inference techniques for estimating the parameters for a classic SIR compartmental model (Bartlett, 1949; Kermack & McKendrick, 1927) consisting of susceptible, infested, and removed states. To allow for intrinsic stochasticity in the spread of OPM, we use an Itô stochastic differential equation (Oksendal, 1995) representation of the SIR model. This is further modified via the introduction of a time‐varying infestation rate, as is necessary to capture the effect of unknown influences such as preventative measures (Dureau et al., 2013). Bayesian inference for the resulting model is complicated by the intractability of the observed data likelihood, and subsequently, the joint posterior distribution of the key quantities of interest (model parameters and dynamic components). We overcome these difficulties via a linear Gaussian approximation of the stochastic SIR model, coupled with a Markov chain Monte Carlo scheme (Fearnhead et al., 2014) for generating samples from the joint posterior. These methods are outlined in Section 2 and detailed in Appendix S1, Sections S1 and S2, for use as a toolbox to apply to other ecological datasets. We use the parameters from the compartmental model to estimate a yearly $R_{0}$ measure for OPM, analogous to the basic reproduction number for a pathogen (Heesterbeek & Dietz, 1996), and estimate the OPM population in 2021.

## 2 METHODS

In this section, we present the observational time series data with a summary of the data collection methods (Section 2.1), the details of the stochastic SIR model (Section 2.2), and an outline of our statistical inference methods (Section 2.3). Further statistical details including the relevant algorithms are set out in Appendix S1 (Sections S1 and S2).

### 2.1 Data

The data in this paper are from Richmond and Bushy Park, collected and processed by The Royal Parks. This is shared with the Forestry Commission on an annual basis to inform the national Oak Processionary Moth Programme (Contingency Plan, 2021). The University of Southampton (GeoData) provide analysis, support, and hold the program data on behalf of the Forestry Commission.

**FIGURE 1:**
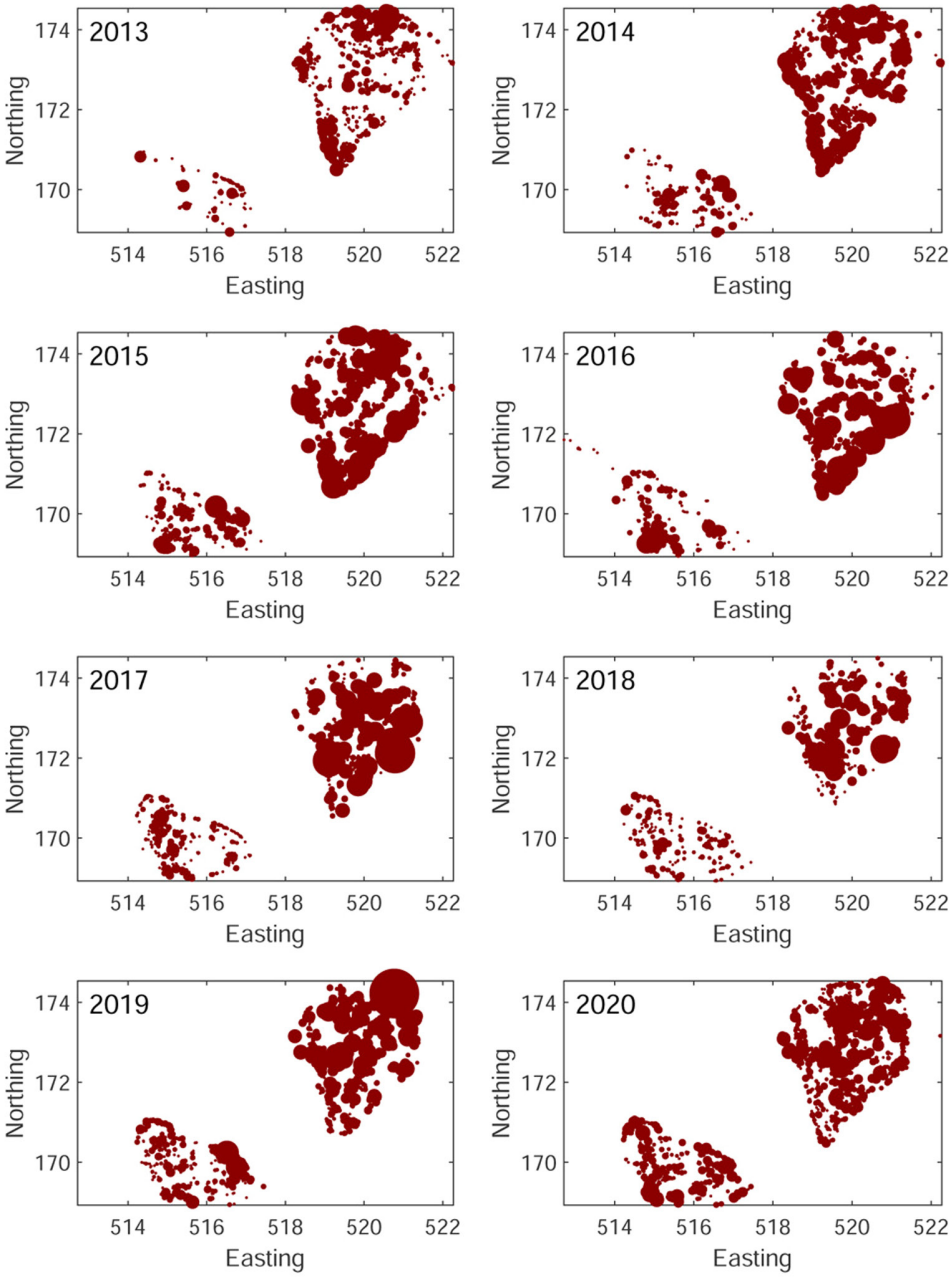
Map of the nests removed from Bushy (bottom left) and Richmond (top right) parks between 2013 and 2020. The area of the marker is proportional to the number of nests removed

The data used in this study were obtained through the recording of OPM nest removals in Richmond and Bushy Parks in South‐West London. For each of the years 2013–2020, it contains (i) the eastings and northings of trees which had nests removed and (ii) the number of nests removed from each tree. The dataset consists of 8470 unique tree locations, with 1767 in Bushy Park and 6703 in Richmond Park. The locations of the trees which had nests removed are shown across the two parks in Figure 1. There are no recordings of the locations of trees, which did not have any nests removed.

**FIGURE 2:**
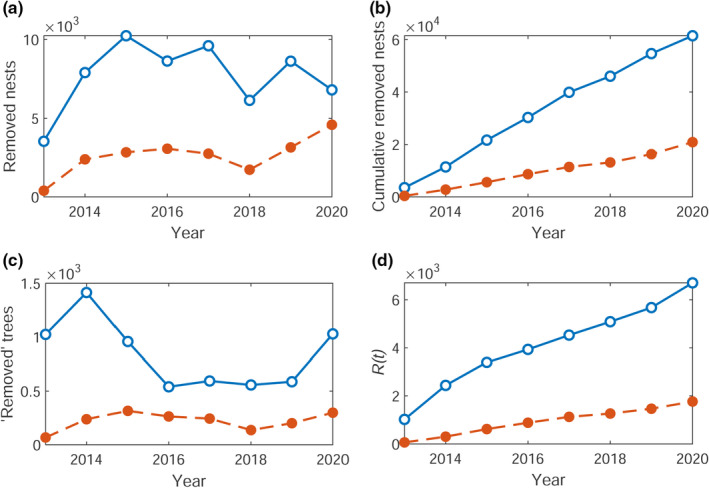
(a) Raw and (b) cumulative number of OPM nests removed from Richmond (blue) and Bushy (orange dashed) parks between 2013 and 2021. The number of (c) raw and (d) cumulative unique trees (described by their eastings and northings) which had nests removed between 2013 and 2021. The cumulative number of trees is the time series R(t)corresponding to the “removed” category in the SIR model (see Section 2.2)

The raw and cumulative time series of the numbers of removed nests are shown in Figure 2(a,b). We count each tree in the year it first had nests removed as one “removal” in the SIR model (see Section 2.2), regardless of how many nests were recorded as removed from this location. The raw and cumulative time series for the number of unique trees which had nests removed are shown in Figure 2(c,d). We use the latter cumulative time series, R(t), as our observational data in the following sections.

### 2.2 Stochastic SIR model

We consider a SIR model (Andersson & Britton, 2000; Kermack & McKendrick, 1927) in which a population of trees of fixed size N is classified into compartments consisting of susceptible (S), infested (I), and removed (R) trees. Although most commonly used in epidemiology, SIR models have previously been used to describe the spread of tree diseases (Parry et al., 2014; Rodriguez‐Quinones & Gordillo, 2019) and invasive species through varying landscapes (Ferrari et al., 2014; Wildemeersch et al., 2019). In our case, susceptible trees are those that have yet to ever be infested with OPM nests and are at risk from the currently infested trees. The time series data we use (see Section 2.1) are observations of the removed category, trees that have previously been infested with OPM nests and have now had these nests removed. A fixed population of trees is appropriate as over the timescale of interest the number of trees born into the S compartment will be sufficiently small to be negligible. Transitions between compartments can be summarized via two pseudo‐reactions of the form

$$S+I\overset{\beta }{⟶}2I,\;I\overset{\gamma }{⟶}R.$$

The first transition describes the “contact” of a currently infested tree with a susceptible tree (in this case, since trees have fixed locations, a probabilistic opportunity for the S to I transition to occur through the dispersal of OPM) and with the net effect resulting in one additional infested tree and one fewer susceptible. The second transition accounts for the currently infested trees moving into the R category as their nests are removed. The parameters β and γ govern the rate of infestation and removal, respectively. The infestation rate for the whole population (assuming one initial infested tree) of trees is thus βN to scale for the number of possible “contacts”. It is clear that transitions should result in discrete changes to the numbers of trees in each state. This most naturally leads to a continuous time, discrete valued Markov jump process (MJP) description of disease dynamics, as detailed in Appendix S1, Section S1 (Ho et al., 2018). We eschew the MJP formalism in favor of a continuous‐valued approximation, formulated as a stochastic differential equation (SDE). This is a pragmatic choice, since the SDE model ultimately leads to a computationally efficient inference scheme, and the model can be easily augmented with additional components, such as time‐varying parameters, which we now describe.

The SDE representation of the standard SIR model can be derived directly from the MJP (see Appendix S1, Section S1). Here, we extend this to include a time‐varying infestation process. Let $X_{t}={\left(S_{t},I_{t},{\tilde{\beta }}_{t}\right)}^{\prime }$ where $S_{t}$and $I_{t}$denote the numbers in each of the states Sand Iat time t≥0and ${\tilde{\beta }}_{t}=\log\beta_{t}$is the (transformed) time‐varying infestation process. Note that the fixed population size gives $R_{t}=N‐S_{t}‐I_{t}$for all t≥0so that the current state of the SIR model is completely described by $X_{t}$. We model ${\tilde{\beta }}_{t}$as a generalized Brownian motion process so that

$$d{\tilde{\beta }}_{t}=\sigma dW_{3,t}$$

and $W_{3,t}$ is a standard one‐dimensional Brownian motion process. Hence, we assume that the log infestation rate evolves according to a random walk in continuous time, with variability controlled by σ. Combining this process with component SDEs describing the dynamics of $S_{t}$ and $I_{t}$ gives the complete SDE description of the SIR model with time‐varying infestation rate as

(1) $$dX_{t}=a\left(x_{t},\theta \right)dt+\sqrt{b(x_{t},\theta )}dW_{t}.$$

Here, $x_{t}={\left(s_{t},i_{t},{\tilde{\beta }}_{t}\right)}^{\prime }$ is the state of the system at time *t*, *θ* = (*γ*, *σ*)′ is the vector of static parameter values, *W_t_* = (*W*_1,_ *_t_*, *W*_2,_ *_t_*, *W*_3,_ *_t_*)′ is a vector of uncorrelated standard Brownian motion processes, and the drift function $a(x_{t},\theta )$and diffusion coefficient $b(x_{t},\theta )$are given by

(2) $$a\left(x_{t},\theta \right)=\left(\begin{array}{c} ‐\exp\left({\tilde{\beta }}_{t}\right)s_{t}i_{t}\\ \exp\left({\tilde{\beta }}_{t}\right)s_{t}i_{t}‐\gamma i_{t}\\ 0 \end{array}\right),\;\;b\left(x_{t},\theta \right)=\left(\begin{array}{ccc} \exp\left({\tilde{\beta }}_{t}\right)s_{t}i_{t} & ‐\exp\left({\tilde{\beta }}_{t}\right)s_{t}i_{t} & 0\\ ‐\exp\left({\tilde{\beta }}_{t}\right)s_{t}i_{t} & \exp\left({\tilde{\beta }}_{t}\right)s_{t}i_{t}+\gamma i_{t} & 0\\ 0 & 0 & \sigma^{2} \end{array}\right).$$

Unfortunately, due to the nonlinear forms of $a(x_{t},\theta )$ and $b(x_{t},\theta )$, the SDE specified by (1) and (2) cannot be solved analytically. We, therefore, replace the intractable analytic solution with a tractable Gaussian process approximation, which is the subject of the next section. The resulting linear noise approximation is subsequently used as the inferential model.

#### 2.2.1 Linear noise approximation

The linear noise approximation (LNA) provides a tractable approximation to the SDE given by (1) and (2). In what follows we give a brief derivation; formal details can be found in Kurtz, (1972) (see also Kampen, 2001; Komorowski et al., 2009).

Consider a partition of $X_{t}$ as

(3) $$X_{t}=\eta_{t}+Z_{t},$$

where $\left\{\eta_{t},t\geq 0\right\}$ is a deterministic process satisfying the ODE

(4) $$\frac{d\eta_{t}}{\mathrm{dt}}=a\left(\eta_{t},\theta \right),\;\;\;\;\;\;\;\;\eta_{0}=x_{0},$$

and $\left\{Z_{t},t\geq 0\right\}$ is a residual stochastic process. The residual process $Z_{t}$ satisfies

$$dZ_{t}=\left\{a\left(x_{t},\theta \right)‐a\left(\eta_{t},\theta \right)\right\}\;dt+\sqrt{b(x_{t},\theta )}\;dW_{t},$$

which will typically be intractable. The assumption that $\left|\right|X_{t}‐\eta_{t}\left|\right|$ is “small” motivates a Taylor series expansion of $a(x_{t},\theta )$ and $b(x_{t},\theta )$ about $\eta_{t}$, with retention of the first two terms in the expansion of a and the first term in the expansion of b. This gives an approximate residual process $\left\{{\hat{Z}}_{t},t\geq 0\right\}$ satisfying

$$d{\hat{Z}}_{t}=H_{t}{\hat{z}}_{t}\;dt+\sqrt{b(\eta_{t},\theta )}\;dW_{t},$$

where $H_{t}$ is the Jacobian matrix with (i,j)th element

$${\left(H_{t}\right)}_{i,j}=\frac{\partial a_{i}\left(\eta_{t},\theta \right)}{\partial \eta_{j,t}}.$$

For the SIR model in (1) and (2), we therefore have

$$H_{t}=\left(\begin{array}{ccc} ‐\exp\left({\tilde{\beta }}_{t}\right)i_{t} & ‐\exp\left({\tilde{\beta }}_{t}\right)s_{t} & ‐\exp\left({\tilde{\beta }}_{t}\right)s_{t}i_{t}\\ \exp\left({\tilde{\beta }}_{t}\right)i_{t} & \exp\left({\tilde{\beta }}_{t}\right)s_{t}‐\gamma & \exp\left({\tilde{\beta }}_{t}\right)s_{t}i_{t}\\ 0 & 0 & 0 \end{array}\right).$$

Given an initial condition ${\hat{Z}}_{0}∼N\left({\hat{z}}_{0},{\hat{V}}_{0}\right)$, it can be shown that ${\hat{Z}}_{t}$ is a Gaussian random variable (see Fearnhead et al., 2014). Consequently, the partition in (3) with $Z_{t}$ replaced by ${\hat{Z}}_{t}$, and the initial conditions $\eta_{0}=x_{0}$ and ${\hat{Z}}_{0}=0$ give

(5) $$X_{t}∼N\left(\eta_{t},V_{t}\right),$$

where $\eta_{t}$ satisfies (4) and $V_{t}$ satisfies

(6) $$\frac{dV_{t}}{\mathrm{dt}}=V_{t}H_{t}^{\prime }+b\left(\eta_{t},\theta \right)+H_{t}V_{t},\;\;\;\;V_{0}=0.$$

Further details on the derivation of (6) are given in Appendix S1, Section S1. Hence, the linear noise approximation is characterized by the Gaussian distribution in (5), with mean and variance found by solving the ODE (ordinary differential equation) system given by (4) and (6). Although this ODE system will typically be intractable, a numerical scheme can be straightforwardly applied.

### 2.3 Bayesian inference

We consider the case in which not all components of the stochastic epidemic model are observed. Moreover, we assume that data points are subject to measurement error, which accounts for mismatch between the latent and observed process, due to, for example, the way in which the data are collected. Observations (on a regular grid) $y_{t},t=0,1,\cdots n$ are assumed conditionally independent (given the latent process) with conditional probability distribution obtained via the observation equation,

(7) $$Y_{t}∼N\left(P^{\prime }x_{t},\sigma_{e}^{2}P^{\prime }x_{t}\right),\;\;\;\;\;\;t=0,1,\ldots ,n$$

where *P* = (1, 1, 0)′. This choice of P is due to the data consisting of observations on the removed state $R_{t}$, which, for a known population size N, is equivalent (in information content) to observing the sum $S_{t}+I_{t}$. Note that the logarithm of the infestation rate process, ${\tilde{\beta }}_{t}$, is completely unobserved. Our choice of observation model is motivated by a Gaussian approximation to a Poisson *Po*(*P*′*x_t_*) distribution, with the role of $\sigma_{e}^{2}$ to allow a decoupling of the mean and variance. Moreover, the assumption of a Gaussian observation model admits a tractable observed data likelihood function, when combined with the LNA (see Section 2.2, Fearnhead et al., 2014; Golightly et al., 2015) as a model for the latent epidemic process $X_{t}$. Details on a method for the efficient evaluation of this likelihood function can be found in Section S2.3 of Appendix S1.

Given data $y=(y_{0},y_{1},\cdots ,y_{n})$ and upon ascribing a prior density π(θ) to the components of *θ* = (*γ*, *σ*, *σ_e_*)′ (augmented to include $\sigma_{e}$), Bayesian inference proceeds via the joint posterior for the static parameters θ and unobserved dynamic process $x=(x_{0},x_{1},\cdots ,x_{n})$. We have that

(8) π(θ,x|y)∝π(θ)π(y|θ)π(x|y,θ),

where π(y|θ) is the observed data likelihood and π(x|y,θ) is the conditional posterior density of the latent dynamic process. Although π(y|θ) and π(x|y,θ) can be obtained in closed form under the LNA, the joint posterior in (8) is intractable. In Appendix S1 Section S2, we describe a Markov chain Monte Carlo scheme for generating (dependent) samples from (8). Briefly, this comprises two steps: (i) the generation of samples $\theta^{\left(1\right)},\cdots ,\theta^{\left(M\right)}$ from the marginal parameter posterior π(θ|y)∝π(θ)π(y|θ) and (ii) the generation of samples $x^{\left(1\right)},\cdots ,x^{\left(M\right)}$ by drawing from the conditional posterior $\pi (x|y,\theta^{\left(i\right)})$, i=1,⋯,M.

Given inferences on the static parameters θ and the latent dynamic process x, we consider the following diagnostics for assessing model fit. The within‐sample predictive density is

(9) $$\pi \left(\tilde{y}|y\right)=\int \int \pi \left(\tilde{y}|x,\theta \right)\pi \left(\theta ,x|y\right)dxd\theta ,$$

and the one step ahead out of sample predictive density is

(10) $$\pi \left(y_{n+1}|y\right)=\int \int \pi \left(y_{n+1}|x_{n+1},\theta \right)\pi \left(x_{n+1}|x_{n},\theta \right)\pi \left(\theta ,x|y\right)dx_{0:n+1}d\theta .$$

Hence, in both cases we properly account for parameter and latent process uncertainty. Although the densities in (9) and (10) will be intractable, we may generate samples via Monte Carlo, see Appendix S1 Section S2 for further details.

## 3 RESULTS

We assume the epidemic time series (see Section 2.1) for the cumulative number of trees with removed nests, R(t), shown in Figure 2(c), can be described by the compartmental SIR model with a time‐varying infestation rate (see Section 2.2). The aim is to estimate the key parameters through the Bayesian inference techniques described in Section 2.3. These are the time‐varying infestation rate per tree, β(t), with corresponding stochastic noise parameter σ describing $d{\tilde{\beta }}_{t}=d\log\left(\beta_{t}\right)=\sigma W_{3,t}$, the removal rate γ, and the observation error $\sigma_{e}$.

Section S2.4 of Appendix S1 provides details of the assumed population sizes for each park, initial numbers for the S (susceptible) and I (currently infected) tree categories and the initial infestation rate, along with the starting parameter values for the MCMC scheme and prior specification. Regarding the latter, we take an independent prior specification for the components of θ, so that $\pi \left(\theta \right)=\pi \left(\gamma \right)\pi \left(\sigma \right)\pi \left(\sigma_{e}\right)$. We then take lognormal LN(1,1) distributions for σ and $\sigma_{e}$, and a lognormal LN $\left(0,0.5^{2}\right)$ distribution for γ. We assume that initial log infestation rate ${\tilde{\beta }}_{0}$ follows a Gaussian N $\left(‐8.5,\;0.5^{2}\right)$ distribution. These choices are motivated by the assumption of a median removal time of around 1 year (95% credible interval: (0.38, 2.66)), and a basic reproduction number at time 0 of $R_{0}=\beta_{0}N/\gamma$ covering a wide range of plausible values. For example, with $N=5\times 10^{3}$ the prior distributions for γ and ${\tilde{\beta }}_{0}$ lead to a 95% credible interval for $R_{0}$ of (0.25,4.1). The initial conditions are chosen based on the increase in the removal category in the first available year, for example, for Richmond Park there were 1414 new trees in the removal category between 2013 and 2014 (new trees that had nests removed in 2014), so we assume this was approximately the number of infested locations in 2013. We investigated several choices for initial conditions and find our results robust to these variations.

### 3.1 Inference results

We ran the MCMC scheme for 10 $\times 10^{3}$ iterations and monitored the resulting chains for convergence. Indicative trace plots can be found in Appendix S1, Figure S1 and suggest that the sampler has adequately explored the parameter space. Additional chains initialized at different starting values (not shown) further confirm convergence.

**FIGURE 3:**
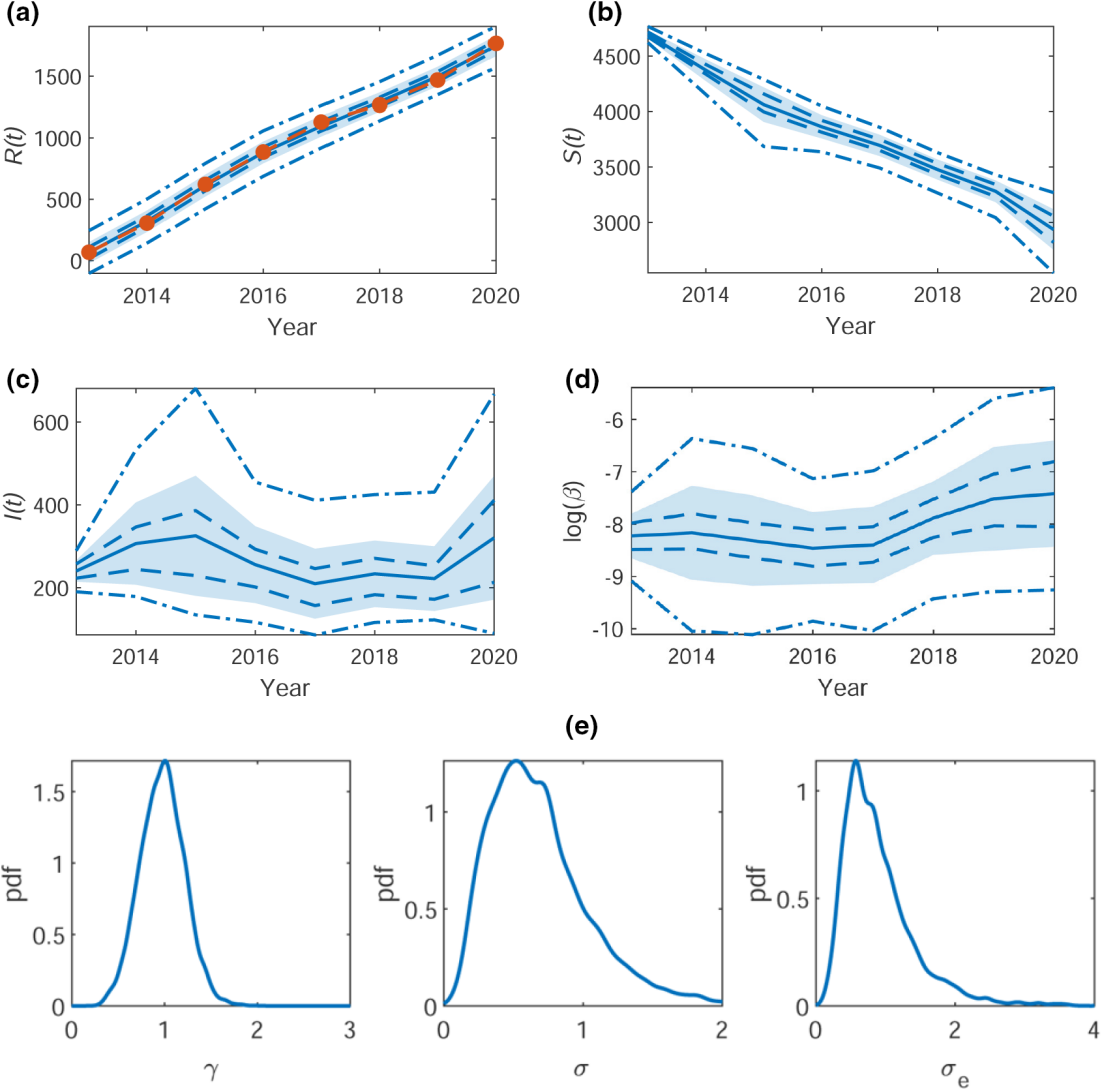
Bushy Park. The within‐sample posteriors for (a) R(t), (b) S(t), (c) I(t), and (d) $\log(\beta_{t})$ with mean (blue solid line) ± one standard deviation (shaded region), the 50% (blue dashed), and the 95% (blue dot‐dashed) credible regions. The observed time series for R(t) is overlaid in (a) (orange dashed). The corresponding (e) posterior densities for the inferred parameters γ (removal rate), σ (noise on ${\tilde{\beta }}_{t}$) and $\sigma_{e}$ (observation error)

**FIGURE 4:**
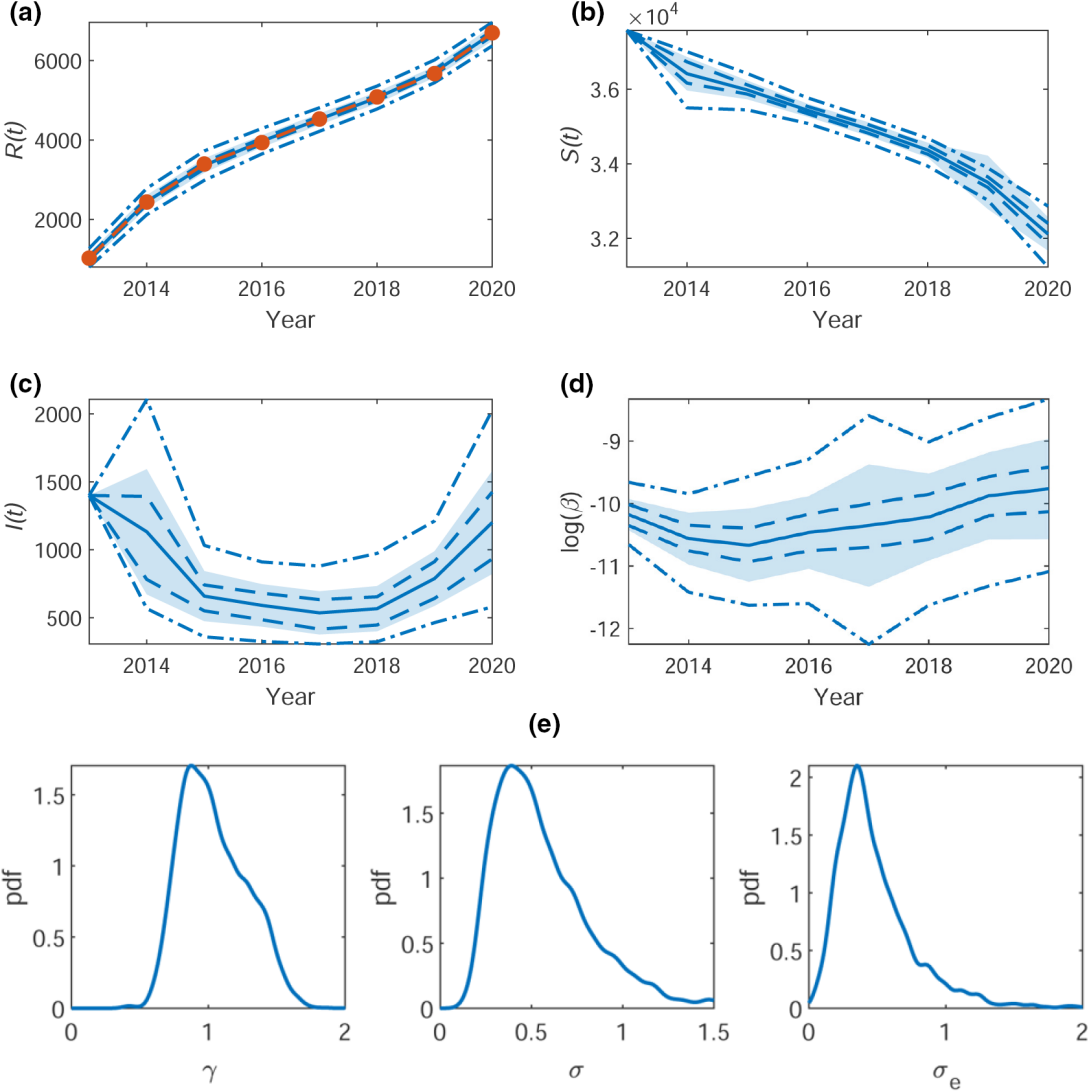
Richmond Park. The within‐sample posteriors for (a) R(t), (b) S(t), (c) I(t), and (d) $\log(\beta_{t})$ with mean (blue solid line) ± one standard deviation (shaded region), the 50% (blue dashed), and the 95% (blue dot‐dashed) credible regions. The observed time series for R(t) is overlaid in (a) (orange dashed). The corresponding (e) posterior densities for the inferred parameters γ (removal rate), σ (noise on ${\tilde{\beta }}_{t}$) and $\sigma_{e}$ (observation error)

From the main MCMC run, we obtain the posterior within‐sample means (with 50% and 95% credible intervals) for R(t), S(t), and I(t), shown in Figures 3 and 4(a–c) for Bushy and Richmond Park, respectively. The logarithmic time‐dependent infestation rate, ${\tilde{\beta }}_{t}=\log\left(\beta \right)$, is shown in Figures 3 and 4(d). For Bushy Park, the logarithmic infestation rate is plausibly constant (given *a posteriori* variance) at ${\tilde{\beta }}_{t}\approx ‐8$, corresponding to an approximate infestation rate of $\beta =3.4\times 10^{‐4}$ and thus for the whole population of Bushy park an infestation rate of βN=1.7. Similarly, for Richmond Park, the infestation rate is plausibly constant with ${\tilde{\beta }}_{t}\approx ‐10$, corresponding to an infestation rate of $\beta =4.5\times 10^{‐5}$ and βN=1.8. Reassuringly, samples from the within‐sample predictive for R(t) are consistent with the data used to fit the model (see panel (a) of Figures 3 and 4).

The posterior density plots of the parameters $\theta =(\gamma ,\sigma ,\sigma_{e})$ are shown in Figures 3 and 4(e), for Bushy and Richmond park, respectively. Pairwise joint posterior densities can be found in Appendix S1, Figure S2. The marginal posterior distribution of γ is centered around γ≈1 for both Bushy and Richmond. The marginal posterior for σ is centered around σ≈0.75 for Bushy and σ≈0.5 for Richmond. The observation error $\sigma_{e}$ is centered around $\sigma_{e}\approx 1$ for Bushy and $\sigma_{e}\approx 0.5$ for Richmond.

### 3.2 Estimation of $R_{0}$

**FIGURE 5:**
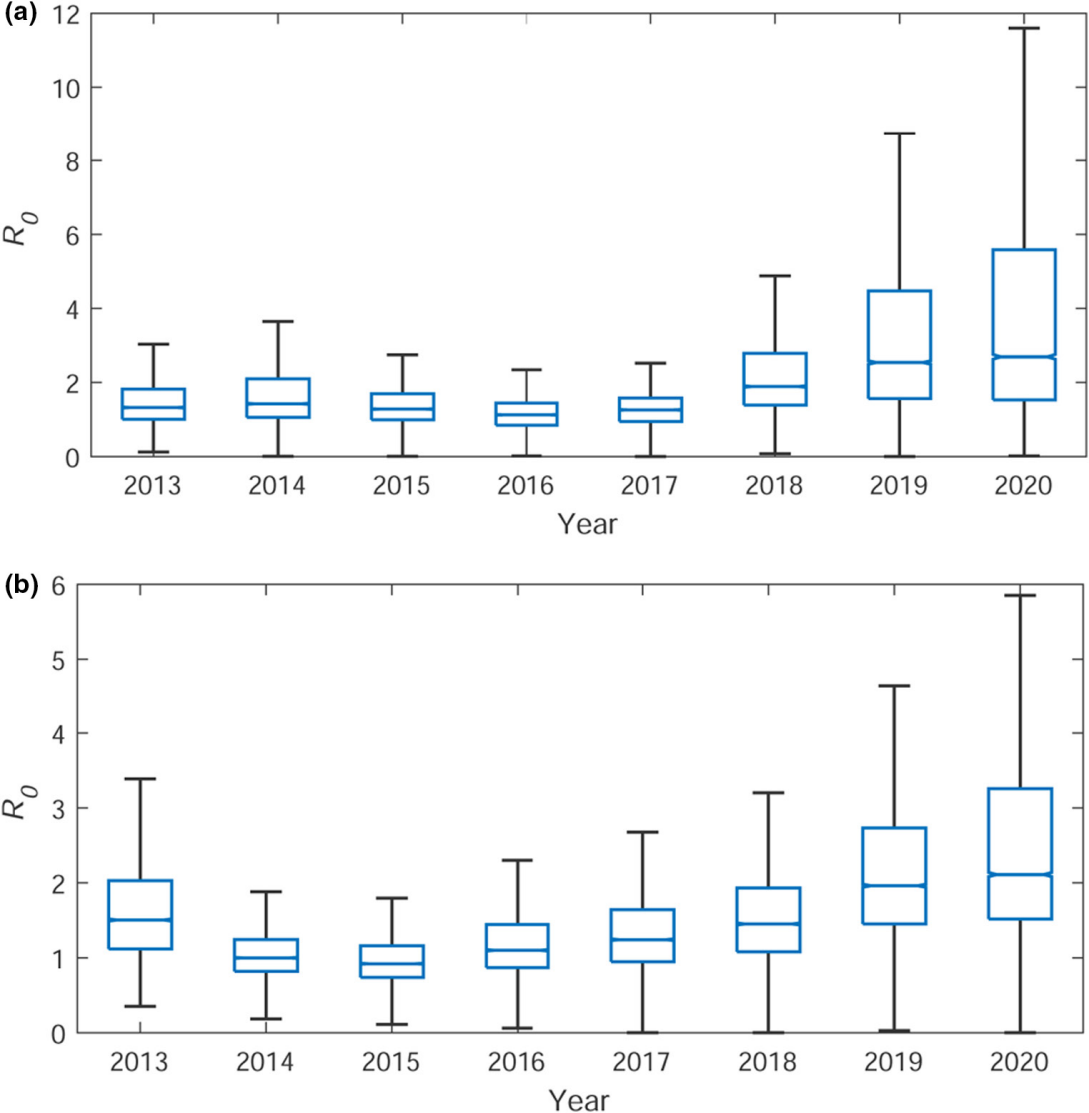
Posterior distributions of $R_{0}\left(t\right)=\beta_{t}N/\gamma$ for (a) Bushy and (b) Richmond Park. The central line indicates the median, with the bottom and top edges of the box showing the 25th and 75th percentiles, respectively. The whiskers extend to the most extreme data points not considered outliers, which are not shown here

From the posterior estimations of ${\tilde{\beta }}_{t}$ for each year and the parameter γ, we can estimate the basic reproduction number $R_{0}$. This gives a measure of the strength of infectivity through the number of trees which become infested as a result of a single infested tree over its infested life time (i.e., the expected number of secondary infestations resulting from a single original infestation). This provides additional information over the parameter β, since $R_{0}$ takes into account the lifetime in which a tree can infest another tree (i.e., the removal rate γ). In a deterministic system, for an epidemic to die out, $R_{0}$ must be less than the threshold value of one. However, in the stochastic case, it is possible for $R_{0}$ to be above one but the epidemic still die out as a result of the stochastic fluctuations. Therefore, it is required that $R_{0}<1$ for the epidemic to shrink, upon averaging over the stochasticity. In a SIR model with a constant infestation rate, β, the basic reproduction number is given by $R_{0}=\beta N/\gamma$. Here, we adapt this to use the time variant infestation rate to get a reproduction number for each of the years between 2013 and 2020, $R_{0}=\beta \left(t\right)N/\gamma$. Box plots showing the posterior distributions of $R_{0}$ for both parks are shown in Figure 5. For both parks $R_{0}$ has been stable, within errors, since 2013 (corresponding to the relatively constant $\beta_{t}$). However, this suggests that $R_{0}$ is still above one, and therefore, the epidemic will continue to propagate in these areas and potentially beyond.

### 3.3 Forward prediction

**FIGURE 6:**
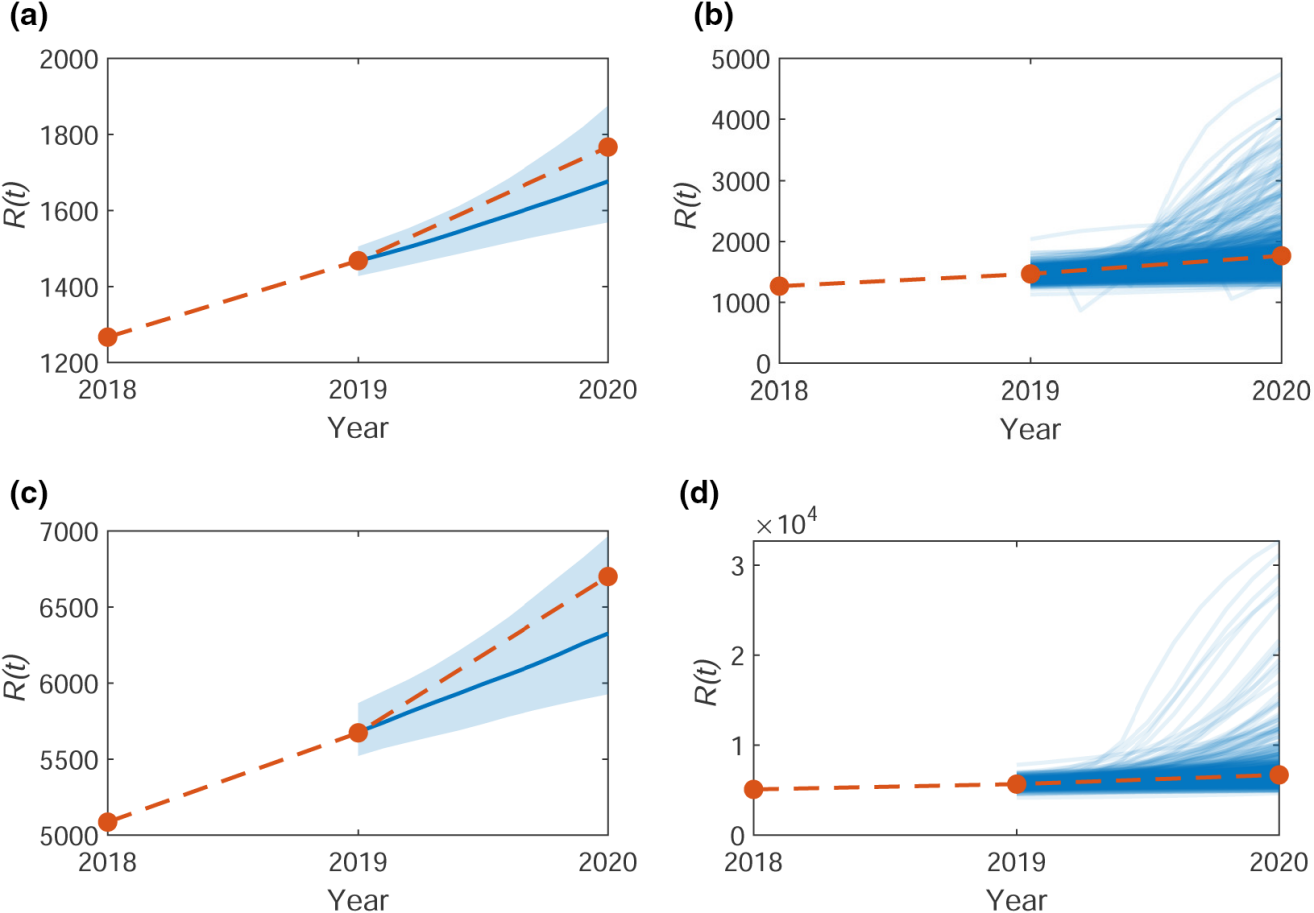
Model predictions for the total number of trees with removed nests up to 2020, R(2020), with median (blue line) for (a, b) Bushy and (c, d) Richmond. In (a) and (c), the shaded area shows the 50% credible region. In (b) and (d), 100 simulations are shown from the forward model. The orange line shows the observed data

Predictions of the spread of OPM are needed to inform control strategies. To test the validity of the SIR model with the inferred parameters from Section 3.1 and thus how well the model can capture future expansions in OPM, we can calculate a one‐year prediction for a known data point. We remove the last data point, R(2020), and re‐infer the parameters for the new shortened observed time series. We then use these parameters to run the model forward ($10\times 10^{3}$) simulations, matching the number of iterations in the MCMC) and obtain an estimate for R(2020). The median predictions with upper and lower quartiles for 1000 runs are shown in Figure 6(a,c) for Bushy and Richmond, respectively. In both cases, the predictive interval captures the observed data. Realizations from 100 forward runs are shown in Figure 6(b,d) to show that results are mostly concentrated around the observed data, with some outliers over‐estimating R(t). One‐step predictions for the whole time series are shown in Appendix S1, Figure S3. This suggests that the available data are sufficient to inform the model to make predictions over these time scales.

**FIGURE 7:**
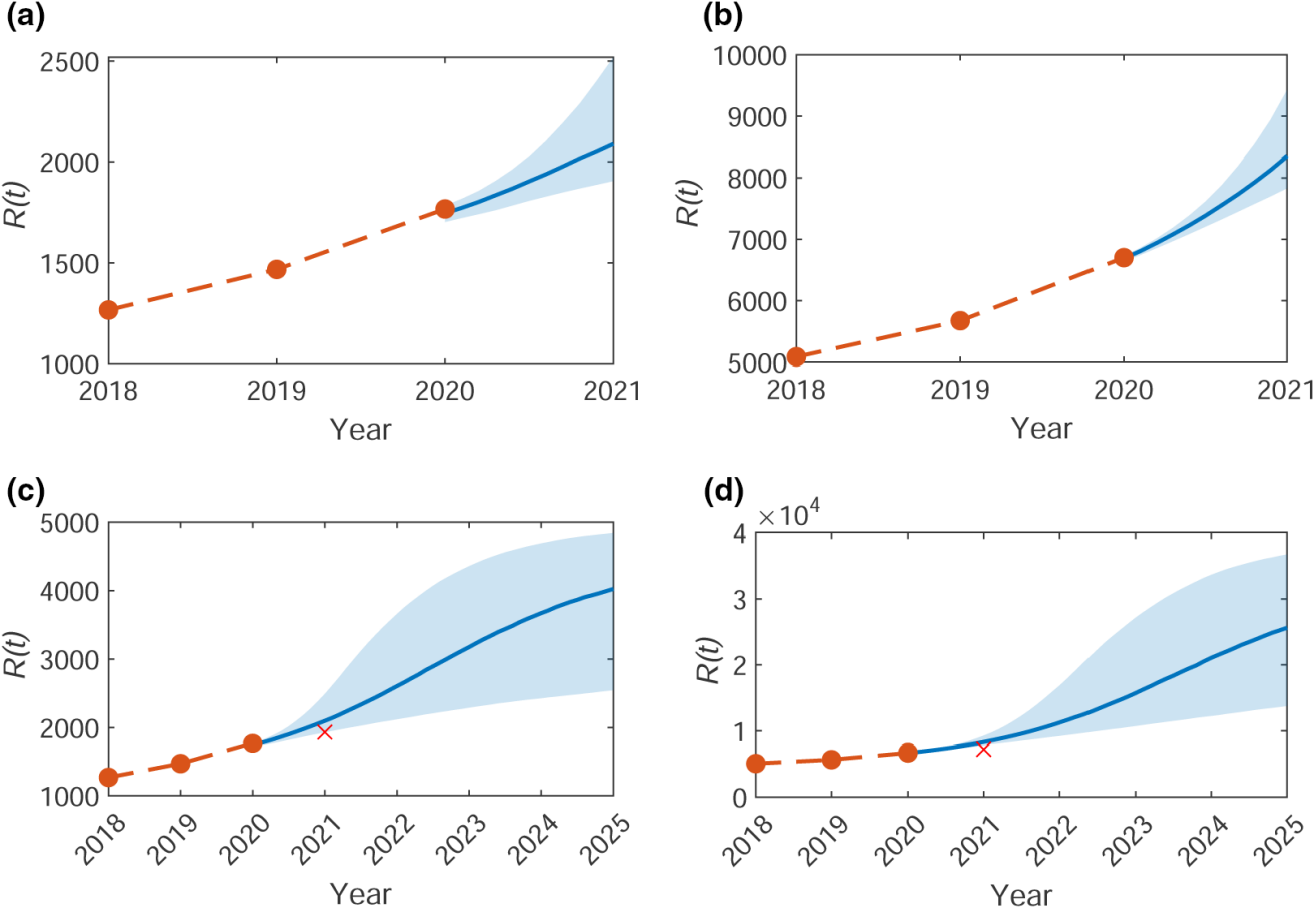
Model predictions for the median (solid blue line) total number of trees with removed nests up to 2021, R(2021), for (a) Bushy and (b) Richmond park, and up to 2025, R(2025), for (c) Bushy and (d) Richmond park. The shaded area shows the 50% credible region. The orange line shows the observed data up to 2020 used to parameterize the model, and the red cross shows the most recent data for 2021

Similarly, we can produce predictions for the number of infested locations in 2021, R(2021). The median predictions with upper and lower quartiles are shown in Figure 7(a,b) for Bushy and Richmond, respectively. This corresponds to an average (median) of 350 new infested locations (lower‐upper quartile estimate range 150–800) in Bushy Park and 1100 (700–2000) in Richmond Park. Since submission of this manuscript, the data for 2021 were recorded, with 167 new infested locations in Bushy and 523 in Richmond Park. This is lower than our mean predicted estimates, which could be due to increased efficacy of the control methods with time. Longer‐term predictions up to the year 2025 are shown in Figure 7(c,d) for Bushy and Richmond, respectively. By this time, R(t) is beginning to saturate due to a depletion of available trees that have not yet previously been infested, that is, the infestation has spread through the whole susceptible population.

## 4 DISCUSSION

Recent modeling work has suggested that the surroundings of the current OPM infestation area in the UK are highly climatically suitable and, therefore, at very high risk from future infestations (Godefroid et al., 2020). Since the government strategy for the containment of OPM relies on targeted control at the boundary of the current infested area, it is crucial to understand and be able to predict the future spread to optimize both the cost and efficacy of these control programs (Contingency Plan, 2021).

We have shown the applicability of a SIR compartmental model with a time‐varying infestation rate to describe the OPM epidemic in the UK between the years 2013 and 2020. Such models have previously been used to describe the spread of tree diseases (Rodriguez‐Quinones & Gordillo, 2019) and invasive species (Ferrari et al., 2014; Wildemeersch et al., 2019). The statistical methodology used is a powerful tool for inferring the parameters of such models from real data and is transferable to other epidemiological and ecological datasets. Previously, similar statistical methodology has been used to describe the spread of infectious diseases (e.g., measles (Cauchemez & Ferguson, 2008) and Ebola (Fintzi et al., 2020)) and the spatial expansion of non‐native plants (Cook et al., 2007), but has not yet been applied to the study of invasive insects.

Our results show, along with previous analysis (Suprunenko et al., 2021), that the spread of OPM is continuing at a stable rate despite the current intervention methods. Correspondingly, we show that the basic reproduction number $R_{0}$ has been above one since 2013. To see a reduction in the OPM population density and to protect the surrounding areas, a reduction of $R_{0}$ to below one would need to be seen. Although the basic reproduction number $R_{0}$ is typically used in the modeling of infectious diseases (Dietz, 1993; Ma, 2020), here it gives an analogous measure for the expected number of infested trees caused by a single currently infested tree through its infested lifetime.

Driven by the nature of the data collected, we chose to make the assumption that the trees with removed nests best represented the removed category in the SIR model. Although not explicitly formulated in this model, we expect that after nest removal and spraying with a biological insecticide, these trees will not be susceptible to future infestation on the short time scales considered here. This limitation of the model could be explored further through assuming this data instead represented the infested category (with the caveat that these trees would not actually be infective to others at the times the data were collected) or by extending the model to an SIRS formulation, which would allow for re‐infestation after a period of immunity.

For simplicity and to be better described by a SIR model, we counted each tree that had undergone nest removal as one removed tree, regardless of how many nests were recorded as being removed from it. However, the defoliation effects and risks to human health from OPM are closely related to nest density (i.e., the numbers of nests per tree) (Jactel et al., 2011). In future work, nest density could be taken into account through a nest density‐dependent infestation rate.

A challenge of modeling OPM and other tree pests and diseases is the lack of a complete inventory oak trees in the UK, representing the susceptible population in our SIR model. This has been previously noted and highlighted as a priority for future data collection by other modeling studies (Cowley et al., 2015). It is of particular importance for future spatial models of OPM, which require an estimate of the distribution of oak trees in the areas of interest.

It is also worth noting that many areas infested with OPM have been undergoing control measures (Contingency Plan, 2021) and so any inferred infestation rates represent the dynamics under these controls, rather than the inherent parameters of the uncontrolled pest spread. In Richmond and Bushy Parks, control measures include the yearly nest removal and limited spraying with a biological insecticide. It would be interesting to conduct a similar analysis on a contained area that had undergone no (or different) control measures to assess the differences in the infestation rates and thus assess the efficacy of the controls. The effect of confounding factors such as the weather, difference in landscapes, and the presence of other pests and parasitoids should also be investigated.

The results from this work can inform the development of future mathematical models for the spread of OPM. These models can be used to identify at‐risk regions (Cowley et al., 2015) and predict the distribution of OPM on a national scale. The development of these models will require further targeted data collection to obtain complete oak tree inventories, as well as data on the population numbers and locations of OPM (or indeed any other invasive insect or pathogen).

## AUTHOR CONTRIBUTIONS

**Laura E. Wadkin** involved in conceptualization (equal); formal analysis (equal); investigation (lead); methodology (equal); project administration (lead); software (equal); visualization (lead); and writing—original draft (lead). **Julia Branson and Andrew Hoppit** involved in writing—review and editing (supporting). **Nicholas G. Parker and Andrew W. Baggaley** involved in conceptualization (equal); funding acquisition (equal); methodology (equal); supervision (equal); and writing—review and editing (equal). **Andrew Golightly** involved in conceptualization (equal); formal analysis (equal); funding acquisition (equal); methodology (equal); software (equal); supervision (equal); and writing—review and editing (equal).

## Supporting information

Supplementary Material

## Data Availability

OPM data: The data used in this paper were collected, processed, and kindly shared by The Royal Parks charity. This data can be provided from The Royal Parks upon reasonable request. Statistical modeling software: all inference schemes and modeling software with example time series are available on the Newcastle University data repository at 10.25405/data.ncl.19292216.
