# Supplementary material for: Inference for epidemic models with time‐varying infection rates: Tracking the dynamics of oak processionary moth in the UK
Source: Ecol Evol. 2022 May 2;12(5):e8871. doi: 10.1002/ece3.8871 (PMC9058805; doi:10.1002/ece3.8871)
Supplement: Supplementary file 1 — Supplementary Material [file ECE3-12-e8871-s001.pdf]

Supplementary information

S1 SIR Model

S1.1 SIR as a Markov Jump Process

Consider a SIR model in which a population of fixed size N is classified into compartments consisting of susceptible (S), infected (I) and removed (R) individuals. Let $X_t = (S_t, I_t)'$ denote the numbers in each state at time $t \geq 0$ and note that $R_t = N - S_t - I_t$ for all $t \geq 0$. The dynamics of $\{X_t, t \geq 0\}$ can be described by a Markov jump process (MJP), that is, a continuous time, discrete valued Markov process. Assuming that at most one event can occur over an infinitesimal time interval $(t, t + \Delta t]$ and that the state of the system at time t is $x_t = (s_t, i_t)'$, the MJP is characterised by probabilities of the form

$$\begin{aligned}
\mathbb{P}(X_{t+\Delta t} = (s_t - 1, i_t + 1)'|x_t, \theta) &= h_1(x_t, \theta)\,\Delta t + o(\Delta t), \\
\mathbb{P}(X_{t+\Delta t} = (s_t, i_t - 1)'|x_t, \theta) &= h_2(x_t, \theta)\,\Delta t + o(\Delta t), \\
\mathbb{P}(X_{t+\Delta t} = (s_t, i_t)'|x_t, \theta) &= 1 - \sum_{i=1}^{2} h_i(x_t, \theta)\,\Delta t + o(\Delta t),
\end{aligned}$$

where $h(x_t, \theta) = (\beta s_t i_t, \gamma i_t)'$ is a hazard function, $\theta = (\beta, \gamma)'$ is a parameter vector containing infestation and removal rates and $o(\Delta t)/\Delta t \to 0$ as $\Delta t \to 0$. The transition probability $\pi(x_t|x_0, \theta)$ governing the dynamics of the MJP over arbitrary time intervals of length t can be shown (van Kampen, 2001) to satisfy the chemical master equation (CME):

$$\frac{d}{dt}\pi(x_t|x_0, \theta) = \sum_{i=1}^{2} \left[h_i(x_t - S^i, \theta)\pi(x_t - S^i|x_0, \theta) - h_i(x_t, \theta)\pi(x_t|x_0, \theta)\right]$$

where S^i denotes the ith column of the stoichiometry matrix

$$S = \begin{pmatrix} -1 & 0 \\ 1 & -1 \end{pmatrix}$$

which encodes the effect of each respective transition on the components of X_t. Although it is possible to evaluate $\pi(x_t|x_0, \theta)$ efficiently (Ho et al., 2018), we eschew the MJP formalism in favour of an approximation whereby X_t is modelled by a stochastic differential equation (SDE).

S1.2 SDE representation

Consider an infinitesimal time interval, $(t, t + dt]$, over which the hazard function $h(x_t, \theta)$ will remain constant almost surely. Let dN_t denote the counting process with components $dN_{1,t}$ and $dN_{2,t}$ containing the number of infestation and removals over this interval. Hence $dN_{i,t}$, is Poisson distributed with rate $h_i(x_t, \theta)dt$. Upon noting that from $dX_t = SdN_t$, it should be clear that

$$\mathrm{E}(dX_t) = S\,h(x_t, \theta)dt, \qquad \mathrm{Var}(dX_t) = S\,\mathrm{diag}\{h(x_t, \theta)\}S'dt.$$

Hence, the Itô Stochastic differential equation (SDE) that best matches the MJP is given by

$$dX_t = S\,h(x_t, \theta)dt + \sqrt{S\,\mathrm{diag}\{h(x_t, \theta)\}S'}\,dW_t,$$

where $W_t = (W_{1,t}, W_{2,t})'$ is a 2-vector of standard Brownian motion and $\sqrt{S\,\mathrm{diag}\{h(x_t, \theta)\}S'}$ is a 2×2 matrix B such that $BB' = S\,\mathrm{diag}\{h(x_t, \theta)\}S'$. Explicitly, we have for the SIR model that

$$S\,h(x_t, \theta) = \begin{pmatrix} -\beta s_t i_t \\ \beta s_t i_t - \gamma i_t \end{pmatrix}, \qquad \sqrt{S\,\mathrm{diag}\{h(x_t, \theta)\}S'} = \begin{pmatrix} \beta s_t i_t & -\beta s_t i_t \\ -\beta s_t i_t & \beta s_t i_t + \gamma i_t \end{pmatrix}.$$

In Section 2.2 in the main text, we discuss extending the model to include a time varying infestation rate.

S1.3 Linear noise approximation

In the main text we set out the linear noise approximation as a tractable approximation to the SIR SDE. This gives an approximate residual process $\{\hat{Z}_t, t \geq 0\}$ satisfying

$$d\hat{Z}_t = H_t \hat{z}_t \, dt + \sqrt{b(\eta_t, \theta)} \, dW_t,$$

(see main text Section 2.2). Given an initial condition $\hat{Z}_0 \sim \mathrm{N}(\hat{z}_0, \hat{V}_0)$, it can be shown that $\hat{Z}_t$ is a Gaussian random variable (see Fearnhead et al., 2014). The solution requires the 3×3 fundamental matrix G_t that satisfies the ODE

$$dG_t = H_t G_t, \qquad G_0 = I_3, \tag{11}$$

where I_d is the identity matrix. We can then let $U_t = G_t^{-1} \hat{Z}_t$ and apply the Itô formula to obtain

$$U_t = G_t^{-1} \sqrt{b(\eta_t, \theta)} dW_t.$$

Leading to

$$U_t = U_0 + \int_0^t G_s^{-1} \sqrt{b(\eta_s, \theta)} dW_s.$$

Linearity and Itô isometry results in

$$U_t | U_0 \sim N \left\{ U_0, \int_0^t G_s^{-1} b(\eta_s, \theta) (G_s^{-1})' ds \right\}.$$

Therefore we have that $\hat{Z}_t | \hat{Z}_0 = \hat{z_0} \sim N(G_t \hat{z_0}, G_t \psi_t G_t')$ with $\psi_t = \hat{V_0} + \int_0^t G_s^{-1} b(\eta_s, \theta)(G_s^{-1})' ds$, an approximating distribution of $X_t \sim N(\eta_t, V_t)$ with $V_t = G_t \psi_t G_t'$ is obtained. The ODE for V_t can be found by the product rule, resulting in

$$\frac{dV_t}{dt} = V_t H_t' + b(\eta_t, \theta) + H_t V_t, \qquad V_0 = 0, \tag{12}$$

main text equation (6).

S2 Bayesian Inference under the LNA

We take the linear noise approximation (LNA) as described in Setion 2.2 of the main text as the inferential model. Although the observed data likelihood can be evaluated efficiently (with details below), the joint posterior is intractable. We therefore consider a Markov chain Monte Carlo (MCMC) scheme for generating samples from the posterior.

S2.1 Posterior exploration via MCMC

Our inference strategy comprises two steps:

1. Generate samples $\theta^{(1)}, \ldots, \theta^{(M)}$ from the marginal parameter posterior $\pi(\theta|y) \propto \pi(\theta)\pi(y|\theta)$.
2. Generate samples $x^{(1)}, \ldots, x^{(M)}$ by drawing from the conditional posterior $\pi(x|y, \theta^{(i)})$, $i = 1, \ldots, M$.

In step 1, we use a Metropolis-Hastings algorithm to draw from $\pi(\theta|y)$. This requires evaluation of the observed data likelihood $\pi(y|\theta)$. Note the factorisation

$$\pi(y|\theta) = \pi(y_0|\theta) \prod_{t=1}^{n} \pi(y_t|y_{0:t}, \theta). \tag{13}$$

Following Fearnhead et al., 2014 (see also Golightly et al., 2015), we evaluate each constituent term in (13) via a forward filter. The forward filter requires the observation equation (main text (7)) to have a linear Gaussian structure, which is not the case as written, due to the variance being a function of the latent state process. Therefore, we run step t of the forward filter with $P'x_{t+1}P$ replaced by $P'\eta_{t+1}P$. That is, the unknown x_{t+1} is replaced by the LNA predictive mean η_{t+1}.

Since the parameters θ remain fixed throughout the calculation of $\pi(y|\theta)$, we drop them from the notation where possible. Define $y_{0:t} = (y_0, \ldots, y_t)'$. Now suppose that $X_0 \sim \mathrm{N}(\mathrm{a}_0, \mathrm{C}_0)$ *a priori*. Algorithm 1 (Supplementary Information Section S2.3) gives the forward filter. This can then be used inside Algorithm 2 (Section S2.3), which uses a random walk Metropolis algorithm to generate (dependent) draws from the marginal parameter posterior $\pi(\theta|y)$. The proposal mechanism requires an innovation variance Ω which can be chosen to maximise mixing efficiency, as measured by say

effective sample size per second. We take $\Omega = k\widehat{\text{Var}}(\theta|y)$ where the posterior variance is estimated from a short pilot run and k is chosen subsequently to give an acceptance rate of around 20–30% (Roberts et al., 2001).

Given samples $\theta^{(1)}, \dots, \theta^{(M)}$ from $\pi(\theta|y)$, we generate samples of the latent process $x^{(i)} \sim \pi(x|y, \theta^{(i)})$, $i = 1, \dots, M$ by noting that these samples can be efficiently generated using a backward sampling algorithm. This requires the covariance between X_t and X_{t+1} (since the former is drawn conditionally on a realisation of the latter) which depends on the 3×3 fundamental matrix G_t. This satisfies the ODE system (11) which can be time-stepped with the ODEs in (4) and (6) (main text) as part of the forward filter. Algorithm 3 (Section S2.3) then gives the backward sampler.

S2.2 Model checking

Given inferences on the static parameters θ and the latent dynamic process x, we consider the following diagnostics for assessing model fit. The within sample predictive density is

$$\pi(\tilde{y}|y) = \int\int \pi(\tilde{y}|x, \theta)\pi(\theta, x|y)dxd\theta \tag{14}$$

and the one step ahead out of sample predictive density is

$$\pi(y_{n+1}|y) = \int\int \pi(y_{n+1}|x_{n+1}, \theta)\pi(x_{n+1}|x_n, \theta)\pi(\theta, x|y)dx_{0:n+1}d\theta. \tag{15}$$

Hence, in both cases we properly account for parameter and latent process uncertainty. Although the densities in (14) and (15) will be intractable, we may generate samples via Monte Carlo. Recall that the inference algorithm described in Section S2.1 gives draws $\{(\theta^{(i)}, x^{(i)}), i = 1, \dots, M\}$. We then generate $\{\tilde{y}^{(i)}, i = 1, \dots, M\}$ via (7), by drawing $\tilde{y}_t^{(i)}$ from a $N(P'x_t^{(i)}, (\sigma_e^2)^{(i)}P'x_t^{(i)})$ distribution, independently for $t = 0, \dots, n$ and $i = 1, \dots, M$. Similarly, we generate samples from $\pi(y_{n+1}|y)$ by first drawing $x_{n+1}^{(i)}$ from $\pi(x_{n+1}|x_n^{(i)}, \theta^{(i)})$, followed by $y_{n+1}^{(i)}$ from $\pi(y_{n+1}|x_{n+1}^{(i)}, \theta^{(i)})$.

S2.3 Algorithms

Algorithm 1 LNA forward filter

1. Initialisation. Compute $\pi(y_0) = \mathrm{N}(y_0\,;\, P'a_0\,,\, P'C_0P + \Sigma_0)$ where $\Sigma_0 = \sigma_e^2 P'a_0P$. The posterior at time $t = 0$ is therefore $X_0|y_0 \sim \mathrm{N}(a_1, C_1)$, where

$$
\begin{aligned}
a_1 &= a_0 + C_0P\left(P'C_0P + \Sigma_0\right)^{-1}\left(y_0 - P'a_0\right) \\
C_1 &= C_0 - C_0P\left(P'C_0P + \Sigma_0\right)^{-1}P'C_0\,.
\end{aligned}
$$

   Store the values of a_1 and C_1.

2. For $t = 0, 1, \dots, n-1$,

   (a) Prior at $t+1$. Initialise the LNA with $\eta_t = a_t$ and $V_t = C_t$. Integrate the ODEs (4) and (6) forward to $t+1$ to obtain η_{t+1} and V_{t+1}.

   (b) One step forecast. Using the observation equation (7), we have that

$$
Y_{t+1}|y_{0:t} \sim \mathrm{N}\left(P'\eta_{t+1}, P'V_{t+1}P + \Sigma_{t+1}\right)
$$

   where $\Sigma_{t+1} = \sigma_e^2 P'\eta_{t+1}P$. Compute the updated marginal likelihood

$$
\begin{aligned}
\pi(y_{0:t+1}) &= \pi(y_{0:t})\pi(y_{t+1}|y_{0:t}) \\
&= \pi(y_{0:t}) \times \mathrm{N}\left(y_{t+1}\,;\, P'\eta_{t+1}\,,\, P'V_{t+1}P + \Sigma_{t+1}\right).
\end{aligned}
$$

   (c) Posterior at $t+1$. Combining the distributions in (a) and (b) gives the joint distribution of X_{t+1} and Y_{t+1} (conditional on $y_{0:t}$) as

$$
\begin{pmatrix} X_{t+1} \\ Y_{t+1} \end{pmatrix} \sim \mathrm{N}\left\{\begin{pmatrix} \eta_{t+1} \\ P'\eta_{t+1} \end{pmatrix}, \begin{pmatrix} V_{t+1} & V_{t+1}P \\ P'V_{t+1} & P'V_{t+1}P + \Sigma_{t+1} \end{pmatrix}\right\}
$$

   and therefore $X_{t+1}|y_{0:t+1} \sim \mathrm{N}(a_{t+1}, C_{t+1})$, where

$$
\begin{aligned}
a_{t+1} &= \eta_{t+1} + V_{t+1}P\left(P'V_{t+1}P + \Sigma_{t+1}\right)^{-1}\left(y_{t+1} - P'\eta_{t+1}\right) \\
C_{t+1} &= V_{t+1} - V_{t+1}P\left(P'V_{t+1}P + \Sigma_{t+1}\right)^{-1}P'V_{t+1}\,.
\end{aligned}
$$

   Store the values of a_{t+1}, C_{t+1}, η_{t+1} and V_{t+1}.

Algorithm 2 Random walk Metropolis algorithm

1. Initialise at $\theta^{(0)}$ in the support of $\pi(\theta|y)$. Set the iteration counter $i = 1$.
2. Propose $\theta^* = \theta^{(i-1)} + \epsilon_i$ where $\epsilon_i \sim \mathrm{N}(0, \Omega)$
3. With probability
$$\alpha(\theta^*|\theta^{(i-1)}) = \min\left\{1, \frac{\pi(\theta^*)\pi(y|\theta^*)}{\pi(\theta^{(i-1)})\pi(y|\theta^{(i-1)})}\right\}$$
put $\theta^{(i)} = \theta^*$ otherwise put $\theta^{(i)} = \theta^{(i-1)}$.
4. If $i = M$ stop otherwise put $i := i + 1$ and go to step 2.

Algorithm 3 LNA backward sampler

1. First draw x_n from $X_n|y \sim \mathrm{N}(a_n, C_n)$.
2. For $t = n-1, n-2, \dots, 0$,
   (a) Joint distribution of X_t and X_{t+1}. Note that $X_t|y_{1:t} \sim \mathrm{N}(a_t, C_t)$. The joint distribution of X_t and X_{t+1} (conditional on $y_{1:t}$) is
$$\begin{pmatrix} X_t \\ X_{t+1} \end{pmatrix} \sim \mathrm{N}\left\{\begin{pmatrix} a_t \\ \eta_{t+1} \end{pmatrix}, \begin{pmatrix} C_t & C_t G'_{t+1} \\ G_{t+1}C_t & V_{t+1} \end{pmatrix}\right\}.$$
   (b) Backward distribution. The distribution of $X_t|X_{t+1}, y_{0:t}$ is $\mathrm{N}(\hat{a}_t, \hat{C}_t)$, where
$$\hat{a}_t = a_t + C_t G'_{t+1} V_{t+1}^{-1}\left(x_{t+1} - \eta_{t+1}\right),$$
$$\hat{C}_t = C_t - C_t G'_{t+1} V_{t+1}^{-1} G_{t+1} C_t.$$
   Draw x_t from $X_t|X_{t+1}, y_{0:t} \sim \mathrm{N}(\hat{a}_t, \hat{C}_t)$.

S2.4 Parameters for inference

		Bushy	Richmond
Total population	N	5000	40000
Initial infested	I_0	240	1400
Initial infestation rate	$\tilde{\beta}_0$	-8.5	-10
Initial model parameters	$\theta_0 = (\gamma_0, \sigma_0, \sigma_e)'$	$(1, 0.5, 1)'$	$(2, 0.5, 1)'$
Observation matrix	P	(1, 1, 0)	
Prior distributions	$\pi(\theta)$	$\log\gamma \sim$N(0, 0.5^2)<br>$\log\sigma \sim$N(1, 1)<br>$\log\sigma_e \sim$N(1, 1)	
Tuning parameter	Σ	$\begin{pmatrix} 0.1 & 0 & 0 \\ 0 & 0.1 & 0 \\ 0 & 0 & 1 \end{pmatrix}$	
Initial conditions mean	a_0	$(N - I_0 - R_0,\ I_0,\ \tilde{\beta}_0)$	
Initial conditions variance	C_0	$\begin{pmatrix} 0 & 0 & 0 \\ 0 & 0 & 0 \\ 0 & 0 & 0.5 \end{pmatrix}$	

Table S1: The parameters used in the inference schemes described in Sections 2.3 and S2.

S3 Additional results figures

The following section contains additional results figures referred to in the main text. We give the trace (Figure S1) and 2D contour plots (Figure S2) for the inferred model parameters. The one-step model predictions for all years are given in Figure S3

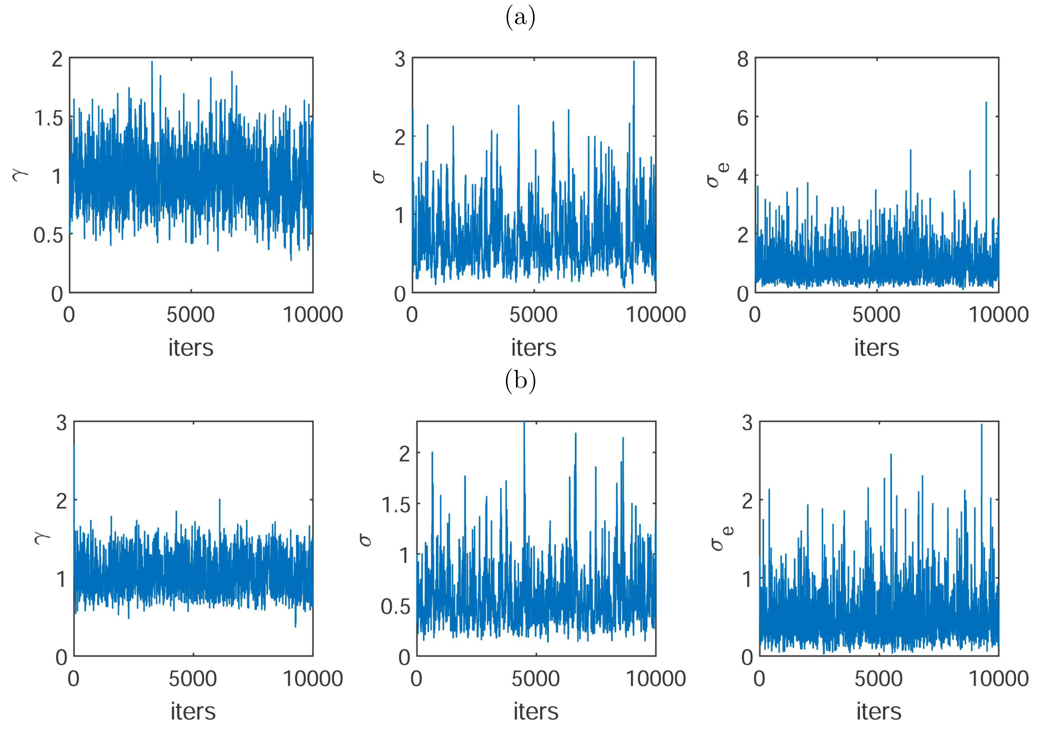

Figure S1: Trace plots for (a) Bushy and (b) Richmond Park.

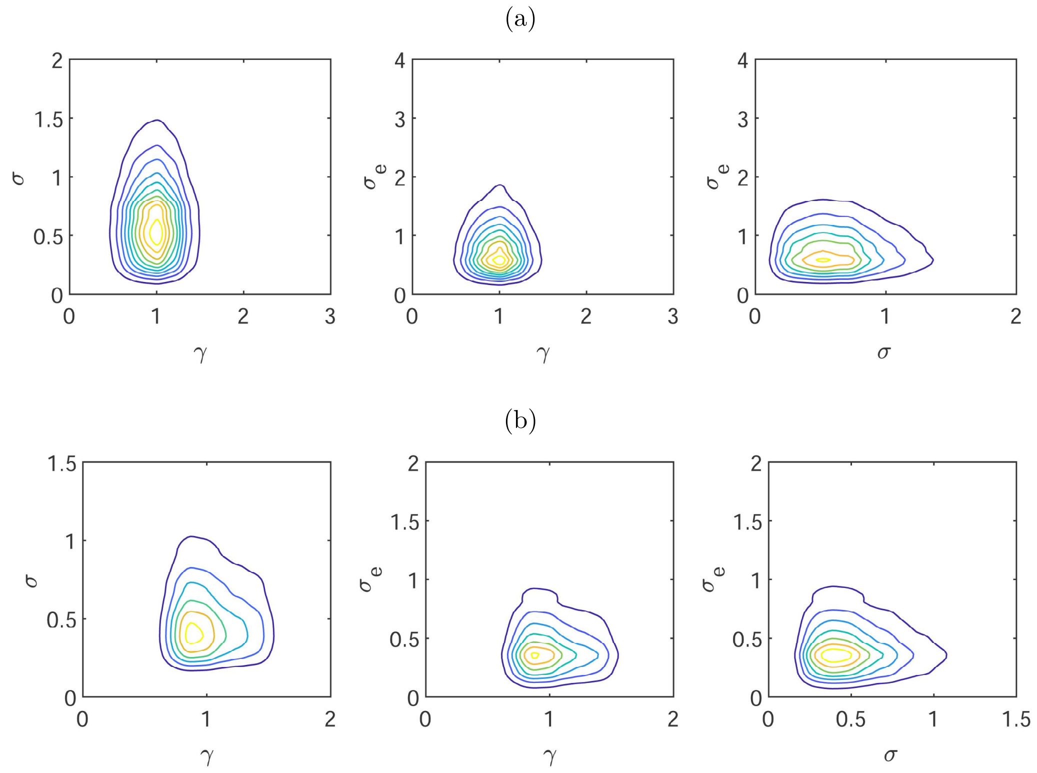

Figure S2: Pairwise joint posterior densities for the parameters γ, σ and σ_e for (a) Bushy and (b) Richmond Park.

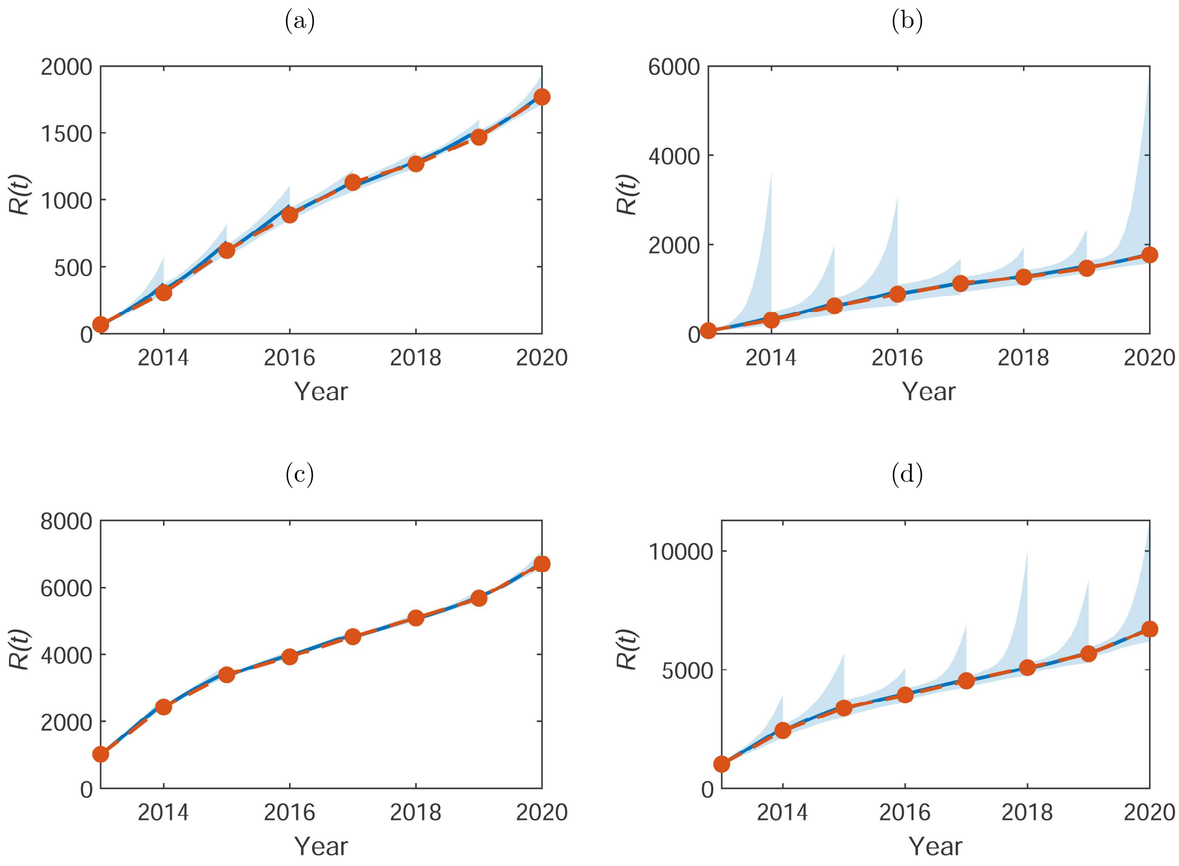

Figure S3: One-step model predictions for the number of removed nests, $R(t)$ for the years 2014–2020, with median (blue line) for (a-b) Bushy and (c-d) Richmond. In (a) and (c) the shaded area shows 50% credible region and in (b) and (d) the 95% credible region. The orange line shows the observed data.

References

Fearnhead, P., Giagos, V. & Sherlock, C. (2014), Inference for reaction networks using the linear noise approximation. Biom, 70: 457-466. https://doi.org/10.1111/biom.12152

Golightly, A., Henderson, D.A. & Sherlock, C. Delayed acceptance particle MCMC for exact inference in stochastic kinetic models. Stat Comput 25, 1039–1055 (2015). https://doi.org/10.1007/s11222-014-9469-x

Ho, L. S. T., Xu, J., Crawford, F.W. et al. Birth/birth-death processes and their computable transition probabilities with biological applications. J. Math. Biol. 76, 911–944 (2018). https://doi.org/10.1007/s00285-017-1160-3

Roberts, G. O. & Rosenthal, J. S., "Optimal scaling for various Metropolis-Hastings algorithms." Statist. Sci. 16 (4) 351 - 367, November 2001. https://doi.org/10.1214/ss/1015346320
